# Comparative Serum Fatty Acid Profiles of Captive and Free-Ranging Cheetahs (*Acinonyx jubatus*) in Namibia

**DOI:** 10.1371/journal.pone.0167608

**Published:** 2016-12-19

**Authors:** Adrian S. W. Tordiffe, Bettina Wachter, Sonja K. Heinrich, Fred Reyers, Lodewyk J. Mienie

**Affiliations:** 1 Department of Paraclinical Sciences, Faculty of Veterinary Science, University of Pretoria, Onderstepoort, South Africa; 2 Department of Research and Scientific Services, National Zoological Gardens of South Africa, Pretoria, South Africa; 3 Centre for Human Metabonomics, Faculty of Natural Sciences, North-West University, Potchefstroom, South Africa; 4 Department of Evolutionary Ecology, Leibniz Institute for Zoo and Wildlife Research, Berlin, Germany; 5 Idexx Laboratories (Pty) Ltd, Woodmead Willow Office Park, Johannesburg, South Africa; Centre for Cellular and Molecular Biology, INDIA

## Abstract

Cheetahs (*Acinonyx jubatus*) are highly specialised large felids, currently listed as vulnerable on the IUCN red data list. In captivity, they are known to suffer from a range of chronic non-infectious diseases. Although low heterozygosity and the stress of captivity have been suggested as possible causal factors, recent studies have started to focus on the contribution of potential dietary factors in the pathogenesis of these diseases. Fatty acids are an important component of the diet, not only providing a source of metabolisable energy, but serving other important functions in hormone production, cellular signalling as well as providing structural components in biological membranes. To develop a better understanding of lipid metabolism in cheetahs, we compared the total serum fatty acid profiles of 35 captive cheetahs to those of 43 free-ranging individuals in Namibia using gas chromatography-mass spectrometry. The unsaturated fatty acid concentrations differed most remarkably between the groups, with all of the polyunsaturated and monounsaturated fatty acids, except arachidonic acid and hypogeic acid, detected at significantly lower concentrations in the serum of the free-ranging animals. The influence of age and sex on the individual fatty acid concentrations was less notable. This study represents the first evaluation of the serum fatty acids of free-ranging cheetahs, providing critical information on the normal fatty acid profiles of free-living, healthy individuals of this species. The results raise several important questions about the potential impact of dietary fatty acid composition on the health of cheetahs in captivity.

## Introduction

Cheetahs (*Acinonyx jubatus*) are kept in many facilities worldwide. Despite obvious improvements in husbandry since cheetahs were first kept in captivity, they still suffer from a range of unusual diseases not typically seen in other large captive felids. These include glomerulosclerosis [1–4], renal amyloidosis [4], lympho-plasmacytic gastritis [2,5,6], veno-occlusive disease [2,7], splenic myelolipomas, cardiac fibrosis [2,4], adrenal cortical hyperplasia [1,2,4,8] with lymphocytic depletion of the spleen [2], pancreatic atrophy [2] as well as several ill-defined disorders of the neurological system [2,9]. Some of these chronic degenerative diseases eventually affect the majority of cheetahs in captivity and are considered to be the primary cause of morbidity and mortality in adult animals [2,10]. In contrast, the incidence of similar conditions in free-ranging cheetahs was found to be very low [10,11]. Although low heterozygosity and the stress of captivity have been suggested as causal factors [8,12], recent studies have started to focus on the contribution of potential dietary factors in the pathogenesis of these diseases [13–15]

There is increasing evidence of the critical role of dietary and circulating fatty acids (FA) in health and disease in various species [16–19]. Besides providing a valuable source of energy, FAs also perform other vital functions in the body, including hormone production, cellular signalling as well as providing structural components of biological membranes. Long-chain polyunsaturated fatty acids (PUFAs), with 20 carbon atoms, are the precursors of eicosanoids, such as prostaglandins, leukotrienes and thromboxanes, which have a wide range of regulatory, autocrine and paracrine effects. PUFAs with 20 to 22 carbon atoms are also the precursors of autacoids, including resolvins, lipoxins and neuroprotectins, which are involved in the active resolution of inflammation [20]. Furthermore, FAs are known to play a role as modulators of gene transcription [21,22].

Given their biological importance, dietary FAs are likely to play a significant role in the health of captive cheetahs. Serum FAs have been shown to provide a good approximation of dietary intake [23–26]. To date few studies have focused on the metabolism of lipids in this species and no clear links have been established between the FA intake and the diseases suffered by cheetahs in captivity. Suspected essential FA deficiencies have been documented in a few cheetah case reports. [27,28]. Currently, serum reference values exist for phospholipid fractions of a selected number of FAs in 28 captive cheetahs from two facilities in the United States [29]. However, the phospholipid FA fraction generally makes up only around 30% of the total serum or plasma FAs and is not necessarily reflective of total serum or plasma FA composition [26] Furthermore, these cheetahs were fed a commercial diet that is only readily available in North America. Given the range of diets fed to captive cheetahs, these values are unlikely to provide reliable serum reference values for cheetahs in other captive facilities.

We hypothesized that the differences in diet, feeding interval and energy expenditure between captive and free-ranging cheetahs result in differences in their serum FA profiles. Captive cheetahs in southern Africa are often fed diets that consist primarily of lean muscle meat from cattle, horses or donkeys, supplemented with a multivitamin/mineral powder. Most facilities adhere to guidelines that recommend feeding meals of between 1.2 and 1.4 kg per day for six days of the week [30]. In contrast, the free-ranging cheetahs have been shown to consume a variety of game species, consisting primarily of small to medium sized antelope and smaller quantities of warthogs, hares and birds [31]. Free-ranging cheetahs also generally feed less frequently, with successful kills only being recorded every 2.6 to 7 days [32,33]. The high proportion of antelope in the free-ranging cheetah diet is expected to result in a lower proportional intake of unsaturated fat, since the unsaturated to saturated fat ratio in ruminant tissues is substantially lower than in monogastric animals. Ruminant muscle tissue typically has an unsaturated to saturated FA ratio of between 0.11 and 0.15, while the ratio in donkey meat is around 1.45 [34–36]. The specific components and organs of prey animals consumed by free-ranging cheetahs should also lead to lower serum PUFA to SFA ratios, as the intra-abdominal fat depots, frequently consumed by free-ranging cheetahs, also have a higher proportion of saturated fat than either intramuscular or subcutaneous adipose tissue [37]. Most African ungulates have substantial intra-abdominal fat reserves, including, mesenteric, omental, perirenal and channel fat. Consumption of whole prey carcasses is therefore likely to result in an increased intake of dietary fat compared to captive diets that are mostly limited to muscle meat and bone [38].

The increased proportional intake of dietary fat, decrease in feeding frequency and increased physical activity in free-ranging compared to captive cheetahs are all predicted to result in enhanced mitochondrial FA oxidation through the lowering of circulating glucose concentrations and insulin:glucagon ratios [39]. During fasting/refeeding cycles and increased levels of exercise, tissue PUFA concentrations have been shown to deplete rapidly in both humans and rats [40,41]. These studies show that most PUFAs, including α-linolenic acid (ALA) and linoleic acid (LA), are preferentially oxidized in periods of exercise or fasting. During refeeding, SFAs and monounsaturated fatty acids (MUFAs), such as palmitic acid and oleic acid, are also more rapidly replaced than any of the PUFAs. Similarly, the concentrations of most plasma PUFAs and MUFAs have been shown to be significantly lower in rats fed a high fat ketogenic diet than in controls [42]. The predicted increase in FA oxidation in free-ranging cheetahs is therefore likely to also skew their serum FA profiles toward lower proportional serum concentrations of PUFAs and MUFAs relative to SFA.

In this study we compared the total serum FA profiles (combined free and bound FAs) of captive cheetahs fed a meat-based diet to those of free-ranging cheetahs feeding on natural prey. Our aim was to investigate our hypothesis that the different feeding habits of free-ranging and captive cheetahs lead to different serum FA profiles and to test two predictions derived from this hypothesis. We expected that free-ranging cheetahs have; 1) lower PUFA:SFA ratios and 2) lower MUFA:SFA ratios than the captive cheetahs. The profiles obtained also provide valuable baseline information with which to evaluate suspected FA deficiencies in captive cheetahs and advance our understanding of lipid metabolism and its impact on the health of this species.

## Materials and Methods

### Ethical and permit considerations

The project was approved by the National Zoological Gardens of South Africa’s Research and Ethics Committee (Project no. P11/07). Research/collecting permits (1846/2013) and (1689/2012) were obtained from the Namibian Ministry of Environment and Tourism and the samples were imported into South Africa with the CITES export (no. 0042838) and import (no. 137670) permits, as well as a veterinary import permit (no. 13/1/1/30/2/10/6-2013/11/002397). Once in South Africa, the samples were transported and stored with the national Threatened or Protected Species (TOPS) ordinary permit (no. 05238).

### Samples

Blood samples were collected from 35 adult captive cheetahs (14 females and 21 males) during their annual health assessments in July 2013 at the AfriCat Foundation near Otjiwarongo in Namibia (Table 1). The cheetahs were fasted for 24 to 36 hours and immobilized via remote injection dart (Daninject, Denmark) with 0.03 mg/kg medetomidine hydrochloride (Medetomidine 20mg/ml, Kyron Laboratories, South Africa) in combination with 1.2 mg/kg zolazepam/teletamine (Zoletil^®^, Virbac, South Africa). These animals were fed a diet consisting mostly of muscle meat from eviscerated and exsanguinated donkeys, supplemented with a multivitamin and mineral powder (Predator Powder ^®^, Healthtech, South Africa).

**Table 1:** Numbers of cheetahs in the categories age, sex and captivity status.

Sex	Age category	Captive	Free-ranging	Total
**Males**	Sub-adults	0	14	14
	Young adults	6	9	15
	Mature adults	8	11	19
	Old adults	7	0	7
	**Total**	**21**	**34**	**55**
**Females**	Sub-adults	0	2	2
	Young adults	4	3	7
	Mature adults	3	4	7
	Old adults	7	0	7
	**Total**	**14**	**9**	**23**

Samples were also collected between July 2012 and May 2013 from 43 free-ranging sub-adult and adult cheetahs (34 males and 9 females) living on commercial farmland in the Khomas and Omaheke districts in central Namibia. They were captured in non-baited box traps, placed at known cheetah marking trees. Once secured in the box traps, they were immobilised by remote intramuscular injection using a combination of 0.06 mg/kg medetomidine hydrochloride (Medetomidine 10 mg/ml, Kyron Laboratories, South Africa) and 3.2 mg/kg ketamine (Ketamine 1G, Kyron Laboratories, South Africa). The ages of the free-ranging cheetahs were estimated by using the key for body size established by [43], using shoulder height, appearance of the mane and physical lesions such as elbow calluses, scars as well as dental wear.

Within 15 to 35 minutes of immobilisation, blood was collected aseptically by different operators, using slightly different methods. In the captive animals, blood was collected from a jugular vein with a 20ml syringe and 18 gauge needle and then transferred into 6ml BD Vacutainer^®^ tubes (Becton, Dickinson and Company, South Africa), while in the free-ranging cheetahs the blood was collected from the cephalic vein directly into the 6ml BD Vacutainer^®^ tubes through a 21 gauge Vacutainer^®^ needle. All the blood samples were allowed to clot on ice in a cooler box. Samples were centrifuged within 4 hours at either 1500g for 10 minutes (captive cheetahs) or within 4 to 12 hours at 400g for 15 minutes (free-ranging cheetahs), after which the serum was pipetted off and immediately frozen at -20°C. The samples were then transported on dry ice to the laboratory in November 2013 and kept at -80°C until analysis in January 2014.

After the sample collection and completion of the health assessments, the captive cheetahs were placed in wooden recovery crates and 0.075mg/kg atipamezole (Antisedan^®^, Zoetis, South Africa) was administered intramuscularly, whereas the free-ranging cheetahs were placed in a padded box and 0.11mg/kg atipamezole (Antisedan^®^, Pfizer, South Africa) was administered intramuscularly. Once the animals had recovered sufficiently from the effects of the anaesthesia in the crates or padded boxes, respectively, the captive cheetahs were released back into their enclosures and monitored intermittently for 24 hours, whereas the free-ranging cheetahs were released at the spot of capture and monitored until they walked away and out of sight. Most of the free-ranging cheetahs were collared with a GPS collar and their movements after release were confirmed to be normal. In all cases they recovered without incident.

### Sample preparation and reagents

After thawing the samples on ice, they were subject to both acidic and alkaline hydrolysis and then extracted into hexane as described by [44] for the analysis of very long-chain fatty acids (VLCFA). The protocol was, however, modified for the additional quantification of long-chain fatty acids (LCFA) by adding an additional stable isotope standard (eicosanoic acid-d_39_) to the internal standard solution at a concentration of 50 μmol/l. Derivatisation was achieved with N-methyl-N-(tert-butyldimethylsilyl) trifluoroacetamide (MTBSTFA) as described by the same authors.

Eicosanoic-d_39_ acid was obtained from Sigma-Aldrich Pty LTD (Kempton Park, South Africa). The VLCFA stable isotope standards: C26:0-d_4_, C24:0-d_4_, C22:0-d_4_, pristanic acid-d_3_ and phytanic acid-d_3_ were obtained from Dr. Herman ten Brink (VU Medical Centre, Amsterdam, The Netherlands) (http://www.vumc.nl/metabool/index.html). Other reagents were obtained from Larodan (Karolinska Institutet Science Park, Retzius väg 8, SE-171 65 Solna, Sweden), Merck Pty LTD (Modderfontein, South Africa) and Regis Technologies, Inc (Morton Grove, IL, USA).

### Gas chromatography-mass spectrometry

The GC-MS analysis of the serum LCFAs and VLCFAs, was conducted on a Hewlett Packard HP6890 series gas chromatography (GC) system with an Agilent 5973N Mass selective detector fitted with an electron ionization (EI) ion source, an Agilent Technologies 7683 autosampler and a 7683B injector. The GC system was fitted with a Phenomenex GC FocusLiner liner for HP, split/splitless, w/wool, single taper, 4 mm ID x 78.5 mm L x 6.3 mm OD (part number AG0 4680) and a CPSil19 capillary column (25 m×0.25 mm×0.20 μm; Varian). The injection system was used in the splitless mode and kept at 300°C. The interface to the mass selective detector was set at 290°C.

Each sample was injected initially for the detection of VLCFAs in the selected-ion monitoring (SIM) mode and a second time for the detection of the LCFAs in scan mode. For the detection of VLCFAs, the GC separation of the analytes was achieved using the following column temperature program: initial temperature 60°C for 1 min, increase to 240°C at a rate of 30°C/min, further increase to 270°C at a rate of 10°C/min, final increase to 300°C at 4°C/min and 5 min isothermal at the latter level. The mass spectrometer was operated at 70 eV in the SIM mode. A dwell time of 100 ms and a relative EM voltage of 400 V higher than that in the scanning mode were chosen for each ion monitored.

For the detection of LCFAs, the GC separation of the analytes was achieved using the following column temperature program: initial temperature 50°C for 1 min, increase to 270°C at a rate of 30°C/min, further increase to 320°C at a rate of 4°C/min and 5 min isothermal at the latter level. The mass spectrometer was operated at 70 eV in the SCAN mode (scan range of 50–650 amu). Helium was used as the carrier gas at a constant flow rate of 1.0 ml/min for both VLCFA analysis and total FA analysis. MS conditions were as follows: EI mode, ion source temperature, 200°C, multiplier voltage, 1.182 V, solvent delay 5 min.

The FAs, were quantified with Agilent MSD ChemStation E02.00 software. A linear regression was used to calibrate the identification and quantification of the FAs. The linearity, lower detection limit and maximum detection limit were determined for all the FAs. A quality control (QC) standard mixture of human control serum was run with each batch. The human control was prepared by aliquoting 100 μl portions of a normal range pooled serum into screw-cap vials. The mean and standard deviation for each QC sample was calculated with a minimum of 20 between-run values. Control values that fell within standard deviations of the mean were considered acceptable.

### Statistical analysis

Three-way ANOVAs were conducted to compare the main and interactive effects of age, sex and captivity on each of the FA concentrations. Cheetahs were classified as sub-adults (< 2 years of age), young adults (2 to 4 years of age), mature adults (5 to 9 years of age) or old cheetahs (> 10 years of age). Data within each subset was tested for normality using the Shapiro-Wilk’s test (p > 0.05). The concentrations of palmitic acid, stearic acid, nonadecanoic acic, arachidic acid, behenic acid, lignoceric acid, pristanic acid, phytanic acid, total SFA, LA, γ-linonenic acid, arachidonic acid (AA), eicosadienoic acid, eicosapentanoic acid, total ω-6, total PUFA and total FA concentrations were normally or near-normally distributed. The concentrations of myristic acid, heptadecanoic acid, cerotic acid, hypogeic acid, palmitoleic acid, oleic acid, total MUFA, SFA:PUFA, ω-6:ω-3 and the desaturase index had to be logarithmically transformed to meet normality requirements for the ANOVA analyses. All analyses were performed with SPSS version 23.0 for Windows (IBM Corporation). Due to the comparison of multiple FAs, a Bonferroni adjustment was applied to the main effects in the ANOVAs, taking into account the number of analyses. This resulted in an adjusted statistical significance level of 0.0018 (0.05/28). For the two-way and three-way interactions, the statistical significance was kept at 0.05. All simple pairwise comparisons were run with a Bonferroni adjustment.

## Results

Analysis of the main effect of captivity status on the various FA concentrations in the ANOVAs resulted in a number of significant differences, as summarised in Table 2. All of the individual serum MUFAs and PUFAs as well as the total MUFA and PUFA concentrations differed between the free-ranging and captive cheetahs (p < 0.0005), with free-ranging cheetahs having lower values for most FAs. Free-ranging cheetahs also had lower total FA concentrations than the captive cheetahs (p = 0.001), whereas total SFA concentrations did not differ. Both the SFA:PUFA and SFA:MUFA ratios were on average more than three-fold higher in free-ranging cheetahs than in captive cheetahs (p < 0.0005). Eicosapentaenoic acid was the only ω-3 PUFA detected in serum of both free-ranging and captive cheetahs. Other ω-3 PUFAs, such as ALA, docosahexaenoic acid (DHA) and eicosatetraenoic acid, were either absent or below the limits of detection. While total ω-3 and ω-6 concentrations were lower in the free-ranging cheetahs (p < 0.0005), the ω-6:ω-3 ratio was not significantly different (p = 0.198).

**Table 2:** Total serum fatty acid concentrations as means and standard deviations (SD) in μmol/L of free-ranging (n = 43) and captive (n = 35) cheetahs. The results of main effect of captivity status in the three-way ANOVAs are shown, comparing the serum fatty acid concentrations of free-ranging and captive cheetahs. For statistical details see supplementary tables A1-D4 in S1 File. SFA = saturated fatty acids, MUFA = monounsaturated fatty acids, PUFA = polyunsaturated fatty acids. Desaturase index = stearic acid/oleic acid. ND = not detected.

Fatty acid (FA)	Abbreviated chemical structure	Free-ranging	Captive	*F*	*p-value*
		Mean (SD)	Mean (SD)		
Myristic acid	C14:0	**32.88** (17.20)	**31.16** (16.19)	0.046	0.83
Palmitic acid	C16:0	**1385.2** (366.0)	**1182.3** (242.7)	3.387	0.07
Heptadecanoic acid	C17:0	**84.67** (42.8)	**45.10** (9.9)	21.020	<0.0005*
Stearic acid	C18:0	**2127.4** (520.3)	**2028.9** (451.4)	0.121	0.729
Nonadecanoic acid	C19:0	**0.03** (0.02)	**0.05** (0.02)	3.147	0.081
Arachidic acid	C20:0	**30.93** (7.5)	**41.05** (9.1)	17.106	<0.0005*
Behenic acid	C22:0	**40.41** (12.6)	**29.77** (4.7)	12.928	0.001*
Lignoceric acid	C24:0	**56.48** (16.8)	**36.48** (6.2)	30.367	<0.0005*
Cerotic acid	C26:0	**0.83** (0.4)	**0.91** (0.4)	2.582	0.113
Pristanic acid		**0.71** (0.8)	**0.089** (0.05)	6.883	0.011
Phytanic acid		**13.41** (8.9)	**2.30** (0.7)	23.746	<0.0005*
**Total SFA**		**3727** (832)	**3398** (676)	1.464	0.231
Hypogeic acid	C16:1ω9	**24.14** (16.8)	**5.87** (6.7)	20.632	<0.0005*
Palmitoleic acid	C16:1ω7	**18.35** (17.4)	**46.97** (19.3)	26.443	<0.0005*
Oleic acid	C18:1ω9	**178.67** (213.3)	**821.44** (362.4)	59.628	<0.0005*
**Total MUFA**		**221.1** (214.9)	**874.3** (374.7)	55.081	<0.0005*
Linoleic acid	C18:2ω6	**294.2** (125.9)	**1049.79** (329.3)	107.985	<0.0005*
γ-Linolenic acid	C18:3ω6	**8.83** (6.6)	**86.58** (58.1)	52.124	<0.0005*
Eicosadienoic acid	C20:2ω6	**0.83** (0.9)	**10.90** (3.4)	247.177	<0.0005*
Dihomo-γ-linolenic acid	C20:3ω6	**ND**	**ND**	-	-
Arachidonic acid	C20:4ω6	**52.19** (17.8)	**35.91** (8.7)	19.845	<0.0005*
**Total ω6**		**357** (137)	**1183** (355)	114.912	<0.0005*
α-Linolenic acid	C18:3ω3	**ND**	**ND**	-	-
Eicosatetraenoic acid	C20:4ω3	**ND**	**ND**	-	-
Eicosapentaenoic acid	C20:5ω3	**5.54** (4.7)	**14.69** (4.6)	36.860	<0.0005*
Docosahexaenoic acid	C22:6ω3	**ND**	**ND**	-	-
**Total ω3**		**5.54** (4.7)	**14.69** (4.6)	36.860	<0.0005*
**Total PUFA**		**363** (140)	**1183** (355)	114.695	<0.0005*
**ω6:ω3**		**281** (462)	**84.0** (24.2)	1.692	0.198
**SFA:PUFA**		**11.15** (2.9)	**3.01** (0.8)	190.34	<0.0005*
**SFA:MUFA**		**22.07** (6.93)	**5.78** (5.1)	106.66	<0.0005*
**Desaturase index**		**0.09** (0.10)	**0.41** (0.17)	62.181	<0.0005*
**Total FA**		**4314** (984)	**5470** (1253)	13.062	0.001*

* p < 0.0018, Bonferroni adjustment 0.05/28 tests.

Other than for captivity status, main effects for age category were significant for γ-linolenic acid F_3,64_ = 3.92, p = 0.01 (Table C2 in S1 File), eicosadienoic acid F_3,64_ = 5.82, p = 0.001 (Table C5 in S1 File), and arachidonic acid F_3,66_ = 3.10, p = 0.03 (Table C3 in S1 File). The γ-linolenic acid concentrations were higher in captive young adult cheetahs (131.48 μmol/L) than in captive mature adults (58.94 μmol/L, p < 0.0005) and captive old adults (74.9 μmol/L, p = 0.001), but the γ-linolenic acid concentrations measured in the various age categories of free-ranging cheetahs did not differ. Arachidonic acid concentrations were lower in sub-adult free-ranging cheetahs (39.87 μmol/L) than in mature adult free-ranging cheetahs (59.50 μmol/L, p = 0.008), but not significantly different from those of young adult free-ranging cheetahs (51.72 μmol/L). Arachidonic acid concentrations did not differ between any of the captive cheetah age categories. Eisosadienoic acid concentrations decreased with age in the captive cheetahs with mean values of 13.62 μmol/L in young adults, 10.16 μmol/L in mature adults and 9.34 μmol/L in old adults. The concentrations differed only between young and mature adults (p = 0.003) as well as young and old adults (p < 0.0005). In contrast, mean eicosadienoic acid concentrations did not differ with age in the free-ranging cheetahs.

In the ANOVAs there were no significant three-way interactions (age, sex and captivity status) for any of the serum FAs measured (Tables A1 –D4 in S1 File). There was only one significant two-way sex*captivity status interaction which was for stearic acid F_1,66_ = 5.09, p = 0.03 (Table A4 in S1 File). The simple main effect of captivity status on stearic acid concentrations was neither significant for males (F_1,66_ = 2.09, p = 0.15), nor for females (F_1,66_ = 1.82, p = 0.18), but stearic acid concentrations tended to be lower in free-ranging females (1843.42 μmol/L) than in free-ranging males (2198.43 μmol/L, F_1,66_ = 3.90, p = 0.053, (data not shown).

Significant two-way captivity status*age interactions were detected for oleic acid F_1,66_ = 5.75, p = 0.019 (Table B3 in S1 File), γ-linolenic acid F_1,64_ = 9.72, p = 0.003 (Table C2 in S1 File), eicosadienoic acid F_1,64_ = 6.80, p = 0.011 (Table C5 in S1 File), SFA:MUFA F_1,66_ = 4.89, p = 0.03 (Table D1 in S1 File) and the desaturase index F_1,66_ = 5.31, p = 0.024 (Table D3 in S1 File). Oleic acid concentrations, eicosadienoic acid concentrations and the desaturase indexes did not differ between the different age categories of either captive or free-ranging cheetahs (data not shown).

## Discussion

In this study, the total serum FA profiles of free-ranging cheetahs contrasted markedly with those in the captive cheetahs, reflecting significant differences in dietary FA composition and/or FA metabolism. Differences in dietary fat intake, dietary FA composition, energy expenditure, and feeding frequency could all potentially contribute to these contrasting lipid profiles. The unsaturated FA concentrations differed most remarkably between the groups, with most of the PUFAs and MUFAs having lower serum concentrations in free-ranging cheetahs and only arachidonic acid and hypogeic acid having lower serum concentration in captive cheetahs. As predicted, both the SFA:PUFA and SFA:MUFA ratios were significantly higher in the free-ranging animals. The influence of age and sex on the individual FA concentrations was less notable.

The higher palmitoleic acid and oleic acid serum concentrations in captive cheetahs may be a reflection of dietary intake of these FAs, because these two MUFAs make up approximately 34% of the FAs in donkey meat [35], whereas their combined proportion of muscular FAs in most game species is below 20% [45]. However, both these MUFAs are also synthesised in the liver by stearoyl coenzyme-A desaturase (SCD) with palmitic and stearic acid as substrates. The regulation of SCD has been shown to have considerable physiologic importance, and alterations in SCD expression and regulation have been implicated in several metabolic diseases in humans and laboratory mice [46]. The plasma ratio of C18:0 (stearic acid) to C18:1 (oleic acid) has been used as a “desaturase index” to measure SCD activity in humans [47]. The desaturase index was more than four-fold higher in the captive cheetahs than in the free-ranging individuals. In SCD1 knockout mice, tissue levels of oleic and palmitoleic acid are reduced, while stearic acid and palmitic acid are increased. Other than genetic mutations, elevated circulating insulin and glucose levels are known to increase SCD expression in the liver [48]. Serum insulin and glucose concentrations have not been adequately evaluated in non-anaesthetised captive or free-ranging cheetahs, but the higher desaturase index in captive cheetahs is consistent with our hypothesis that these animals potentially have higher circulating glucose and insulin levels due to more regular feeding and a higher dietary protein intake.

The differences in serum PUFA concentrations between free-ranging and captive cheetahs are also consistent with our hypothesis, because these unsaturated FAs occur at lower serum concentrations in the free-ranging than in captive cheetahs, primarily due to lower dietary intake. We suggest that the preferential oxidation of PUFAs in periods of increased exercise or fasting in the free-ranging individuals could also potentially contribute towards these differences. Arachidonic acid was the only PUFA for which concentrations were higher in the free-ranging cheetahs. This again may reflect differences in dietary intake, as AA makes up a larger proportion (7.63% to 9.3%) of the intramuscular FAs in wild game species such as the springbok (*Antidorcas marsupialis*) [49] than in donkey muscle meat (1.65% to 2.09%) [36]. A greater consumption of organ meat by free-ranging compared to captive cheetahs may also result in a higher proportional intake of AA [50]. Arachidonic acid can be synthesised from LA, however, similar to domestic cats, the activity of the Δ-6 desaturase enzyme, responsible for catalysing the first step of this pathway, is likely to be very low in cheetahs [29,51]. PUFAs are particularly prone to oxidative rancidity and the peroxidation of AA in stored carcass meat could also potentially contribute to lower serum concentrations in captive cheetahs. Domestic cats, fed a diet high in LA, with adequate levels of AA and antioxidants, were found to have significantly lower plasma AA levels at day 140 of the trial, than cats fed a diet containing lower levels of LA [52]. Generally the carcass meat is not stored for 140 days at most captive cheetah facilities, but it is possible that some peroxidation of the long-chain FAs takes place in storage as no antioxidants are added to the meat until just before it is fed to the animals.

The serum concentrations of ω-3 FAs were low in both the captive and free-ranging cheetahs, making up only 0.13% and 0.27% of the total serum FAs, respectively (calculated from Table 2). Omega-3 FAs, such as ALA, DHA and eicosatetraenoic acid, reported in serum or plasma of captive cheetahs [29] and domestic cats [53], were either absent or below the limits of detection. The reason for the absence of DHA in the plasma of both captive and free-ranging cheetahs is potentially shown in an study by Pawlosky *et al* in which they demonstrated that deuterium labelled LA and ALA administered to cats results in their livers producing deuterium labelled long-chained FAs up to adrenic acid (22:4ω-6) and docosapentaenoic acid (22:5ω-3), but not DHA [54]. Deuterium labelled DHA and several other long-chained FAs were, however, detected in the brain tissue of the cats, suggesting that the docosapentaenoic acid is transported from the liver to the brain, and probably the retina, where it is then further metabolised to DHA and where it is critically required for normal function [55]. The very low concentrations or absence of these essential ω-3 FAs in the serum of healthy free-living cheetahs is thus a clear indication that their circulating concentrations should not be used to evaluate dietary deficiencies in this species.

Heptadecanoic acid, also known as margaric acid, is a SFA with an uneven number of carbon atoms and is normally found at low concentrations in the adipose tissue and milk fat of ruminants. The mean serum concentration of this SFA was higher in free-ranging compared to captive cheetahs. Recently, the importance of this FA was demonstrated in another captive hypercarnivore, the bottlenose dolphin (*Tursiops truncates*) [56]. Low serum heptadecanoic acid concentrations were associated with elevated serum concentrations of known markers of metabolic syndrome, including serum ferritin, glucose, insulin and triglycerides in the dolphins. A change in the fish species provided in the dolphin diet, with higher heptadecanoic acid content, resulted in a normalization of these markers. Two other FAs that correlated negatively with the markers of metabolic syndrome in dolphins were AA and behenic acid. Both of these FAs were also detected at significantly lower serum concentrations in captive cheetahs. Captive cheetahs are not known to suffer from metabolic syndrome, although it has been suggested that the glomerulosclerosis lesions commonly found in these animals may be caused by mild but chronic elevations in blood glucose concentrations [1]. It is, nevertheless, interesting that this SFA, presumed to be non-essential in the diet, had such a dramatic metabolic effect in another obligatory carnivore.

The higher serum concentrations of pristanic and phytanic acids in the free-ranging cheetahs are likely to result from the greater consumption of ruminant prey. Phytanic acid (3,7,11,15-teramethylhexadecanoic acid) is produced from phytol, the side chain of chlorophyll, by the micro-organisms present in the ruminant gastrointestinal tract [57]. Most saturated VLCFAs are components of sphingolipids. The C24 sphingomyelin, which incorporates lignoceric acid, is particularly abundant in liver and kidney tissue [58]. Therefore, higher serum concentrations of lignoceric and behenic acid in the free-ranging cheetahs may reflect their higher dietary intake of these organ meats.

The reasons for the age differences in AA in free-ranging cheetahs, and γ-linolenic and eicosadienoic acid in captive cheetahs, is not clear. The captive cheetahs all received the same diet, regardless of age, and therefore these difference are more likely to involve small age related changes in endogenous synthesis and/or oxidation of these FA rather than differences in dietary intake in this group. Similar differences were not observed in the free-ranging cheetahs and therefore other factors related to captivity are potentially involved. Despite apparent differences in the prey preferences of free-ranging male and female cheetahs [31], none of the serum FAs differed significantly between the sexes, suggesting that the dietary fatty acid intake is reasonably uniform in free-ranging animals. It is therefore unlikely that the lower serum AA concentrations in free-ranging sub-adults are the result of lower dietary intake of this FA. The absence of any sub-adult cheetahs in the captive cheetah group leaves a gap in our data and it is possible that future studies that include captive cheetahs in this age category may find lower AA serum concentrations than in older captive animals.

In this study, we documented the total serum FA profiles of 43 apparently healthy free-ranging cheetahs in Namibia. The unusual diseases suffered by captive cheetahs are rarely reported in free-ranging cheetahs [3,10,11]. Thus, the FA profiles of free-ranging cheetahs provide a reasonable set of reference values against which the profiles of captive individuals can be assessed. The potential links between the diseases suffered by captive cheetahs and the FA composition of their diet, however, still remain unclear. Future studies will need to focus on evaluating the associations between the incidence and/or severity of disease and the various serum FAs, as well as the clinical effects of dietary manipulations and/or FA supplementation. The relative effects of extended feeding intervals and increased physical activity on serum FAs in captive cheetahs will also need to be assessed. Free-ranging cheetahs rarely scavenge and consume most of their prey within a few hours after a kill. Fats consumed under these conditions have little time to become rancid and the higher intake of the oxidatively more stable SFAs would result in a reduced need for extensive antioxidant mechanisms to deal with peroxidised FAs in this species. The extent and impact of dietary FA peroxidation should therefore also be assessed in future studies.

A limitation of our study was the lack of control over the fasting period prior to sampling in the free-ranging cheetahs. Although no bait was used in their capture traps, it is possible that some of the cheetahs may have fed within a few hours before immobilization and sampling. During the postprandial period, the non-esterified fatty acids (NEFAs) generally decline in response to rising insulin levels, while the triacylglycerol lipid fractions increase slightly or remain stable in healthy individuals [59]. Since the NEFA fraction makes up only approximately 6% of the total serum FAs [26], the postprandial variation in total serum FAs is likely to be small. Nevertheless, it would be valuable to assess the changes in serum FAs during the postprandial period in healthy captive cheetahs fed on different diets.

An additional potential limitation in our study was the differences in serum separation and sample storage times prior to analysis in the captive versus the free-ranging cheetahs. Although circulating NEFA fractions potentially increase in blood and plasma after 48 hours at 4°C [60], storage of various plasma lipid classes, including triglyceride, cholesterol ester, phospholipid and NEFA fractions at -20°C for a year, was shown to have minimal effect on stability even without the use of nitrogen storage [61]. In a recent review, Metherel and Stark concluded that plasma/serum storage at 4°C for less than 6 days, -20°C for one to three years and -80°C for up to 10 years would not likely result in significant changes in PUFA concentrations. The effects of delayed blood cell separation on total serum or plasma FA concentrations have not to our knowledge been evaluated. However, a delay of 24 hours before separation in samples kept at 4°C, resulted in an increase in NEFA concentrations of between 0% and 8% in plasma [62,63] and 10% in serum [62]. These increases are most like due to the action of lipolytic enzymes which result in a FA shift from the bound fractions to free FA pool. Such changes are not likely to affect the combined/total plasma or serum FA concentrations and therefore the differences in sample handling, prior to storage, would be expected to have a minimal effect on our results. The impact of different sample handling procedures on total serum or plasma FAs under field conditions should nevertheless be investigated in future studies.

In conclusion, we found marked differences in the serum FAs of captive versus free-ranging Namibian cheetahs. These differences are likely to largely reflect differences in dietary fat intake and composition, but the increased physical activity and extended fasting intervals experienced by free-ranging cheetahs may also play a role. The serum profiles suggest that free-ranging cheetahs consume significantly more saturated fat from ruminant species and less unsaturated fat compared to captive cheetahs. This study represents the first evaluation of the serum FAs of free-ranging cheetahs, providing critical information on the normal FA profiles of free-living, healthy individuals of this species. The results raise several important questions about the potential impact of dietary FAs composition on the health of cheetahs in captivity.

## Supporting Information

S1 File Tables A1 to D4, showing ANOVA Type III summaries for serum fatty acids, log transformed fatty acids or fatty acid ratios for the categories age, sex and captivity status. (DOCX)
