# Supplementary material for: Comparative Serum Fatty Acid Profiles of Captive and Free-Ranging Cheetahs (Acinonyx jubatus) in Namibia
Source: PLoS One. 2016 Dec 19;11(12):e0167608. doi: 10.1371/journal.pone.0167608 (PMC5167222; doi:10.1371/journal.pone.0167608)
Supplement: S1 File — (DOCX) [file pone.0167608.s001.docx]

**S1 File. Tables A1 to D4 showing ANOVA Type III summaries for serum fatty acids, log transformed fatty acids or fatty acid ratios for the categories age, sex and captivity status.**

**Table A1. Log of myristic acid.**

| **Source** | **Sum of squares** | ***df*** | **Mean square** | ***F*** | ***p*** |
| --- | --- | --- | --- | --- | --- |
| Sex | 0.05 | 1 | 0.05 | 1.50 | 0.23 |
| Captivity status | 0.002 | 1 | 0.002 | 0.05 | 0.83 |
| Age | 0.04 | 3 | 0.01 | 0.41 | 0.75 |
| Sex : captivity status | 0.03 | 1 | 0.03 | 0.95 | 0.33 |
| Sex : age | 0.07 | 3 | 0.02 | 0.61 | 0.61 |
| Captivity status : age | 0.04 | 1 | 0.04 | 1.10 | 0.30 |
| Sex : captivity status : age | 0.005 | 1 | 0.005 | 0.14 | 0.71 |
| Error | 2.35 | 66 | 0.04 |  |  |

**Table A2. Palmitic acid.**

| **Source** | **Sum of squares** | ***df*** | **Mean square** | ***F*** | ***p*** |
| --- | --- | --- | --- | --- | --- |
| Sex | 87393.47 | 1 | 87393.47 | 1.13 | 0.29 |
| Captivity status | 262270.44 | 1 | 262270.44 | 3.39 | 0.07 |
| Age | 494513.62 | 3 | 164837.87 | 2.13 | 0.11 |
| Sex : captivity status | 96582.81 | 1 | 96582.81 | 1.25 | 0.27 |
| Sex : age | 236986.21 | 3 | 78995.40 | 1.02 | 0.39 |
| Captivity status : age | 26919.06 | 1 | 26919.06 | 0.35 | 0.56 |
| Sex : captivity status : age | 21182.95 | 1 | 21182.95 | 0.27 | 0.60 |
| Error | 4956155.34 | 64 | 77439.93 |  |  |

**Table A3. Log of heptadecanoic acid.**

| **Source** | **Sum of squares** | ***df*** | **Mean square** | ***F*** | ***p*** |
| --- | --- | --- | --- | --- | --- |
| Sex | 0.003 | 1 | 0.003 | 0.14 | 0.71 |
| Captivity status | 0.64 | 1 | 0.64 | 27.19 | <0.0005 |
| Age | 0.08 | 3 | 0.03 | 1.18 | 0.33 |
| Sex : captivity status | 0.04 | 1 | 0.04 | 1.68 | 0.20 |
| Sex : age | 0.03 | 3 | 0.009 | 0.40 | 0.76 |
| Captivity status : age | 0.02 | 1 | 0.02 | 1.003 | 0.32 |
| Sex : captivity status : age | 0.000 | 1 | 0.000 | 0.02 | 0.89 |
| Error | 1.55 | 66 | 0.023 |  |  |

**Table A4. Stearic acid.**

| **Source** | **Sum of squares** | ***df*** | **Mean square** | ***F*** | ***p*** |
| --- | --- | --- | --- | --- | --- |
| Sex | 267646.45 | 1 | 267646.45 | 1.25 | 0.27 |
| Captivity status | 25910.22 | 1 | 25910.23 | 0.12 | 0.73 |
| Age | 1113937.84 | 3 | 371312.61 | 1.73 | 0.17 |
| Sex : captivity status | 1091495.53 | 1 | 1091495.53 | 5.09 | 0.03 |
| Sex : age | 557534.62 | 3 | 185844.87 | 0.87 | 0.46 |
| Captivity status : age | 23763.73 | 1 | 23763.73 | 0.11 | 0.74 |
| Sex : captivity status : age | 253899.76 | 1 | 253899.76 | 1.18 | 0.28 |
| Error | 14164964.94 | 66 | 214620.68 |  |  |

**Table A5. Nonadecanoic acid.**

| **Source** | **Sum of squares** | ***df*** | **Mean square** | ***F*** | ***p*** |
| --- | --- | --- | --- | --- | --- |
| Sex | 6.100E-6 | 1 | 6.100E-6 | 0.02 | 0.90 |
| Captivity status | 0.001 | 1 | 0.001 | 3.15 | 0.08 |
| Age | 0.001 | 3 | 0.000 | 0.63 | 0.60 |
| Sex : captivity status | 0.001 | 1 | 0.001 | 1.46 | 0.23 |
| Sex : age | 0.003 | 3 | 0.001 | 2.44 | 0.07 |
| Captivity status : age | 0.000 | 1 | 0.000 | .75 | 0.39 |
| Sex : captivity status : age | 3.734E-6 | 1 | 3.734E-6 | 0.009 | 0.93 |
| Error | 0.027 | 65 | 0.000 |  |  |

**Table A6. Arachidic acid.**

| **Source** | **Sum of squares** | ***df*** | **Mean square** | ***F*** | ***p*** |
| --- | --- | --- | --- | --- | --- |
| Sex | 97.28 | 1 | 97.2 | 1.52 | 0.22 |
| Captivity status | 1097.78 | 1 | 1097.78 | 17.11 | <0.0005 |
| Age | 502.96 | 3 | 167.65 | 2.61 | 0.06 |
| Sex : captivity status | 1.13 | 1 | 1.13 | 0.02 | 0.89 |
| Sex : age | 168.55 | 3 | 56.18 | 0.88 | 0.49 |
| Captivity status : age | 121.01 | 1 | 121.01 | 1.89 | 0.17 |
| Sex : captivity status : age | 29.54 | 1 | 29.54 | 0.46 | 0.50 |
| Error | 4171.46 | 65 | 64.18 |  |  |

**Table A7. Behenic acid.**

| **Source** | **Sum of squares** | ***df*** | **Mean square** | ***F*** | ***p*** |
| --- | --- | --- | --- | --- | --- |
| Sex | 158.20 | 1 | 158.20 | 1.58 | 0.21 |
| Captivity status | 1296.98 | 1 | 1296.98 | 12.93 | 0.001 |
| Age | 320.06 | 3 | 106.69 | 1.06 | 0.37 |
| Sex : captivity status | 75.53 | 1 | 75.53 | 0.75 | 0.39 |
| Sex : age | 77.03 | 3 | 25.68 | 0.26 | 0.86 |
| Captivity status : age | 69.32 | 1 | 69.32 | 0.70 | 0.41 |
| Sex : captivity status : age | 1.50 | 1 | 1.50 | 0.02 | 0.90 |
| Error | 6621.55 | 66 | 100.33 |  |  |

**Table A8. Lignoceric acid.**

| **Source** | **Sum of squares** | ***df*** | **Mean square** | ***F*** | ***p*** |
| --- | --- | --- | --- | --- | --- |
| Sex | 289.50 | 1 | 289.50 | 1.64 | 0.21 |
| Captivity status | 5338.86 | 1 | 5338.86 | 30.27 | <0.0005 |
| Age | 225.31 | 3 | 75.10 | 0.43 | 0.74 |
| Sex : captivity status | 180.67 | 1 | 180.66 | 1.02 | 0.32 |
| Sex : age | 122.23 | 3 | 40.74 | 0.23 | 0.88 |
| Captivity status : age | 201.61 | 1 | 201.61 | 1.13 | 0.29 |
| Sex : captivity status : age | 30.04 | 1 | 30.04 | 0.17 | 0.68 |
| Error | 11642.03 | 66 | 176.40 |  |  |

**Table A9. Log of cerotic acid.**

| **Source** | **Sum of squares** | ***df*** | **Mean square** | ***F*** | ***p*** |
| --- | --- | --- | --- | --- | --- |
| Sex | 0.06 | 1 | 0.06 | 1.58 | 0.21 |
| Captivity status | 0.09 | 1 | 0.09 | 2.58 | 0.11 |
| Age | 0.07 | 3 | 0.02 | 0.66 | 0.58 |
| Sex : captivity status | 0.06 | 1 | 0.06 | 1.60 | 0.21 |
| Sex : age | 0.03 | 3 | 0.01 | 0.25 | 0.86 |
| Captivity status : age | 0.08 | 1 | 0.08 | 2.28 | 0.14 |
| Sex : captivity status : age | 3.542E-5 | 1 | 3.542E-5 | 0.001 | 0.98 |
| Error | 2.29 | 66 | 0.04 |  |  |

**Table A10. Pristanic acid.**

| **Source** | **Sum of squares** | ***df*** | **Mean square** | ***F*** | ***p*** |
| --- | --- | --- | --- | --- | --- |
| Sex | 0.37 | 1 | 0.37 | 0.92 | 0.34 |
| Captivity status | 2.73 | 1 | 2.73 | 6.88 | 0.01 |
| Age | 0.02 | 3 | 0.008 | 0.02 | 0.996 |
| Sex : captivity status | 0.69 | 1 | 0.69 | 1.74 | 0.19 |
| Sex : age | 0.52 | 3 | 0.17 | 0.43 | 0.73 |
| Captivity status : age | 0.02 | 1 | 0.02 | 0.04 | 0.85 |
| Sex : captivity status : age | 0.17 | 1 | 0.17 | 0.43 | 0.52 |
| Error | 26.188 | 66 | 0.40 |  |  |

**Table A11. Phytanic acid.**

| **Source** | **Sum of squares** | ***df*** | **Mean square** | ***F*** | ***p*** |
| --- | --- | --- | --- | --- | --- |
| Sex | 55.55 | 1 | 55.55 | 1.26 | 0.27 |
| Captivity status | 1046.24 | 1 | 1046.24 | 23.75 | <0.0005 |
| Age | 27.4778 | 3 | 9.16 | 0.21 | 0.89 |
| Sex : captivity status | 99.47 | 1 | 99.47 | 2.26 | 0.14 |
| Sex : age | 53.09 | 3 | 17.70 | 0.40 | 0.75 |
| Captivity status : age | 23.63 | 1 | 23.63 | 0.54 | 0.47 |
| Sex : captivity status : age | 14.04 | 1 | 14.04 | 0.32 | 0.57 |
| Error | 2907.92 | 66 | 44.06 |  |  |

**Table A12. Total saturated fatty acids.**

| **Source** | **Sum of Squares** | ***df*** | **Mean Square** | ***F*** | ***p-value*** |
| --- | --- | --- | --- | --- | --- |
| Sex | 147719.10 | 1 | 147719.10 | 0.26 | 0.62 |
| Captivity status | 852466.25 | 1 | 852466.25 | 1.46 | 0.23 |
| Age | 1803622.56 | 3 | 601207.52 | 1.03 | 0.38 |
| Sex : captivity status | 1911091.90 | 1 | 1911091.92 | 3.28 | 0.08 |
| Sex : age | 1161957.06 | 3 | 387319.02 | 0.67 | 0.58 |
| Captivity status : age | 125341.31 | 1 | 125341.31 | 0.22 | 0.64 |
| Sex : captivity status : age | 411364.68 | 1 | 411364.68 | 0.71 | 0.40 |
| Error | 38424406.25 | 66 | 582187.98 |  |  |

**Table B1. Log of hypogeic acid.**

| **Source** | **Sum of squares** | ***df*** | **Mean square** | ***F*** | ***p*** |
| --- | --- | --- | --- | --- | --- |
| Sex | 0.002 | 1 | 0.002 | 0.01 | 0.91 |
| Captivity status | 3.80 | 1 | 3.80 | 26.12 | <0.0005 |
| Age | 0.57 | 3 | 0.19 | 1.30 | 0.28 |
| Sex : captivity status | 0.11 | 1 | 0.11 | 0.72 | 0.40 |
| Sex : age | 0.32 | 3 | 0.11 | 0.72 | 0.54 |
| Captivity status : age | 0.000 | 1 | 0.000 | 0.001 | 0.97 |
| Sex : captivity status : age | 0.001 | 1 | 0.001 | 0.008 | 0.93 |
| Error | 9.61 | 66 | 0.146 |  |  |

**Table B2. Log of palmitoleic acid.**

| **Source** | **Sum of squares** | ***df*** | **Mean square** | ***F*** | ***p*** |
| --- | --- | --- | --- | --- | --- |
| Sex | 0.13 | 1 | 0.13 | 1.56 | 0.22 |
| Captivity status | 2.28 | 1 | 2.28 | 26.44 | <0.0005 |
| Age | 0.56 | 3 | 0.19 | 2.18 | 0.10 |
| Sex : captivity status | 0.005 | 1 | 0.005 | 0.06 | 0.80 |
| Sex : age | 0.12 | 3 | 0.04 | 0.45 | 0.72 |
| Captivity status : age | 0.31 | 1 | 0.31 | 3.57 | 0.06 |
| Sex : captivity status : age | 0.15 | 1 | 0.15 | 1.70 | 0.20 |
| Error | 5.68 | 66 | 0.09 |  |  |

**Table B3. Log of oleic acid.**

| **Source** | **Sum of squares** | ***df*** | **Mean square** | ***F*** | ***p*** |
| --- | --- | --- | --- | --- | --- |
| Sex | 0.03 | 1 | 0.03 | 0.37 | 0.55 |
| Captivity status | 4.76 | 1 | 4.76 | 59.63 | <0.0005 |
| Age | 0.27 | 3 | 0.09 | 1.14 | 0.34 |
| Sex : captivity status | 0.02 | 1 | 0.02 | 0.28 | 0.60 |
| Sex : age | 0.08 | 3 | 0.03 | 0.32 | 0.81 |
| Captivity status : age | 0.46 | 1 | 0.46 | 5.75 | 0.02 |
| Sex : captivity status : age | 0.002 | 1 | 0.002 | 0.03 | 0.86 |
| Error | 5.27 | 66 | 0.08 |  |  |

**Table B4. Log of total monounsaturated fatty acids.**

| **Source** | **Sum of squares** | ***df*** | **Mean square** | ***F*** | ***p*** |
| --- | --- | --- | --- | --- | --- |
| Sex | 0.02 | 1 | 0.02 | 0.34 | 0.56 |
| Captivity status | 3.69 | 1 | 3.69 | 55.08 | <0.0005 |
| Age | 0.22 | 3 | 0.07 | 1.09 | 0.36 |
| Sex : captivity status | 0.02 | 1 | 0.02 | 0.29 | 0.60 |
| Sex : age | 0.05 | 3 | 0.02 | 0.25 | 0.86 |
| Captivity status : age | 0.38 | 1 | 0.38 | 5.63 | 0.02 |
| Sex : captivity status : age | 0.005 | 1 | 0.005 | 0.08 | 0.78 |
| Error | 4.42 | 66 | 0.07 |  |  |

**Table C1. Linoleic acid.**

| **Source** | **Sum of squares** | ***df*** | **Mean square** | ***F*** | ***p*** |
| --- | --- | --- | --- | --- | --- |
| Sex | 5185.32 | 1 | 5185.32 | 0.09 | 0.77 |
| Captivity status | 6260935.79 | 1 | 6260935.79 | 107.99 | <0.0005 |
| Age | 367890.24 | 3 | 122630.08 | 2.12 | 0.11 |
| Sex : captivity status | 1287.51 | 1 | 1287.51 | 0.02 | 0.88 |
| Sex : age | 66710.72 | 3 | 22236.91 | 0.38 | 0.77 |
| Captivity status : age | 48995.39 | 1 | 48995.39 | 0.85 | 0.36 |
| Sex : captivity status : age | 19386.66 | 1 | 19386.66 | 0.33 | 0.57 |
| Error | 3826669.47 | 66 | 57979.84 |  |  |

**Table C2. γ-Linolenic acid.**

| **Source** | **Sum of squares** | ***df*** | **Mean square** | ***F*** | ***p*** |
| --- | --- | --- | --- | --- | --- |
| Sex | 8.18 | 1 | 8.18 | 0.006 | 0.94 |
| Captivity status | 70228.17 | 1 | 70228.17 | 52.12 | <0.0005 |
| Age | 15831.48 | 3 | 5277.16 | 3.92 | 0.01 |
| Sex : captivity status | 185.21 | 1 | 185.21 | 0.14 | 0.71 |
| Sex : age | 4528.86 | 3 | 1509.62 | 1.12 | 0.35 |
| Captivity status : age | 13094.97 | 1 | 13094.97 | 9.72 | 0.003 |
| Sex : captivity status : age | 1916.84 | 1 | 1916.84 | 1.42 | 0.24 |
| Error | 86228.23 | 64 | 1347.32 |  |  |

**Table C3. Arachidonic acid.**

| **Source** | **Sum of squares** | ***Df*** | **Mean square** | ***F*** | ***p*** |
| --- | --- | --- | --- | --- | --- |
| Sex | 212.29 | 1 | 212.29 | 1.21 | 0.28 |
| Captivity status | 3475.42 | 1 | 3475.42 | 19.85 | <0.0005 |
| Age | 1628.26 | 3 | 542.75 | 3.10 | 0.03 |
| Sex : captivity status | 314.18 | 1 | 314.18 | 1.79 | 0.19 |
| Sex : age | 73.53 | 3 | 24.51 | 0.14 | 0.94 |
| Captivity status : age | 69.27 | 1 | 69.27 | 0.40 | 0.53 |
| Sex : captivity status : age | 88.40 | 1 | 88.40 | 0.505 | 0.48 |
| Error | 11558.469 | 66 | 175.128 |  |  |

**Table C4. Eicosapentaenoic acid.**

| **Source** | **Sum of squares** | ***df*** | **Mean square** | ***F*** | ***p*** |
| --- | --- | --- | --- | --- | --- |
| Sex | 7.24 | 1 | 7.24 | 0.32 | 0.58 |
| Captivity status | 840.29 | 1 | 840.29 | 36.86 | <0.0005 |
| Age | 18.29 | 3 | 6.10 | 0.27 | 0.85 |
| Sex : captivity status | 7.40 | 1 | 7.40 | 0.33 | 0.57 |
| Sex : age | 42.61 | 3 | 14.20 | 0.62 | 0.60 |
| Captivity status : age | 5.60 | 1 | 5.60 | 0.25 | 0.62 |
| Sex : captivity status : age | 4.00 | 1 | 4.00 | 0.18 | 0.68 |
| Error | 1504.57 | 66 | 22.80 |  |  |

**Table C5. Eicosadienoic acid.**

| **Source** | **Sum of squares** | ***df*** | **Mean square** | ***F*** | ***p*** |
| --- | --- | --- | --- | --- | --- |
| Sex | 2.49 | 1 | 2.49 | 0.55 | 0.46 |
| Captivity status | 1109.67 | 1 | 1109.67 | 247.18 | <0.0005 |
| Age | 78.33 | 3 | 26.11 | 5.82 | 0.001 |
| Sex : captivity status | 9.23 | 1 | 9.23 | 2.06 | 0.16 |
| Sex : age | 6.07 | 3 | 2.02 | 0.45 | 0.72 |
| Captivity status : age | 30.51 | 1 | 30.51 | 6.80 | 0.01 |
| Sex : captivity status : age | 0.22 | 1 | 0.22 | 0.05 | 0.82 |
| Error | 287.32 | 64 | 4.49 |  |  |

**Table C6. Total polyunsaturated fatty acids.**

| **Source** | **Sum of squares** | ***df*** | **Mean square** | ***F*** | ***p*** |
| --- | --- | --- | --- | --- | --- |
| Sex | 2847.55 | 1 | 2847.55 | .043 | 0.84 |
| Captivity status | 7669397.09 | 1 | 7669397.09 | 114.70 | <0.0005 |
| Age | 520747.09 | 3 | 173582.36 | 2.60 | 0.06 |
| Sex : captivity status | 4674.69 | 1 | 4674.69 | 0.07 | 0.79 |
| Sex : age | 94969.23 | 3 | 31656.41 | 0.47 | 0.70 |
| Captivity status : age | 122883.20 | 1 | 122883.20 | 1.84 | 0.18 |
| Sex : captivity status : age | 6735.47 | 1 | 6735.47 | 0.10 | 0.75 |
| Error | 4413275.90 | 66 | 66867.82 |  |  |

**Table C7. Total ω-6 fatty acids.**

| **Source** | **Sum of squares** | ***df*** | **Mean square** | ***F*** | ***p*** |
| --- | --- | --- | --- | --- | --- |
| Sex | 3141.89 | 1 | 3141.89 | 0.048 | 0.83 |
| Captivity status | 7509681.84 | 1 | 7509681.84 | 114.91 | <0.0005 |
| Age | 514695.13 | 3 | 171565.04 | 2.63 | 0.06 |
| Sex : captivity status | 4310.06 | 1 | 4310.06 | 0.07 | 0.80 |
| Sex : age | 95455.41 | 3 | 31818.47 | 0.49 | 0.69 |
| Captivity status : age | 121229.43 | 1 | 121229.43 | 1.86 | 0.18 |
| Sex : captivity status : age | 7067.86 | 1 | 7067.86 | 0.11 | 0.74 |
| Error | 4313210.09 | 66 | 65351.67 |  |  |

**Table C8. Log of serum ω-6:ω-3 fatty acid ratios.**

| **Source** | **Sum of squares** | ***df*** | **Mean square** | ***F*** | ***p*** |
| --- | --- | --- | --- | --- | --- |
| Sex | 0.28 | 1 | 0.28 | 1.54 | 0.22 |
| Captivity status | 0.31 | 1 | 0.31 | 1.692 | 0.198 |
| Age | 0.23 | 3 | 0.08 | 0.43 | 0.74 |
| Sex : captivity status | 0.15 | 1 | 0.15 | 0.85 | 0.36 |
| Sex : age | 0.09 | 3 | 0.03 | 0.17 | 0.92 |
| Captivity status : age | 0.08 | 1 | 0.08 | 0.44 | 0.51 |
| Sex : captivity status : age | 0.01 | 1 | 0.01 | 0.04 | 0.85 |
| Error | 11.890 | 66 | 0.18 |  |  |

**Table D1. Log of saturated fatty acid:monounsaturated fatty acid ratios (SFA:MUFA).**

| **Source** | **Sum of squares** | ***df*** | **Mean square** | ***F*** | ***p*** |
| --- | --- | --- | --- | --- | --- |
| Sex | 96.27 | 1 | 96.27 | 3.06 | 0.09 |
| Captivity status | 3297.78 | 1 | 3297.78 | 104.98 | <0.0005 |
| Age | 180.77 | 3 | 60.26 | 1.92 | 0.14 |
| Sex : captivity status | 56.10 | 1 | 56.10 | 1.79 | 0.19 |
| Sex : age | 148.84 | 3 | 49.61 | 1.58 | 0.20 |
| Captivity status : age | 153.65 | 1 | 153.65 | 4.89 | 0.03 |
| Sex : captivity status : age | 70.83 | 1 | 70.83 | 2.26 | 0.14 |
| Error | 2073.36 | 66 | 31.42 |  |  |

**Table D2. Log of saturated fatty acid:polyunsaturated fatty acid ratios (SFA:PUFA).**

| **Source** | **Sum of squares** | ***df*** | **Mean square** | ***F*** | ***p*** |
| --- | --- | --- | --- | --- | --- |
| Sex | 0.01 | 1 | 0.01 | 0.75 | 0.39 |
| Captivity status | 2.86 | 1 | 2.86 | 190.34 | <0.0005 |
| Age | 0.07 | 3 | 0.02 | 1.45 | 0.24 |
| Sex : captivity status | 0.04 | 1 | 0.04 | 2.64 | 0.11 |
| Sex : age | 0.02 | 3 | 0.005 | 0.35 | 0.79 |
| Captivity status : age | 0.02 | 1 | 0.019 | 1.29 | 0.26 |
| Sex : captivity status : age | 5.870E-7 | 1 | 5.870E-7 | 0.000 | 1.00 |
| Error | 0.99 | 66 | 0.015 |  |  |

**Table D3. Log of the desaturase index.**

| **Source** | **Sum of squares** | ***df*** | **Mean square** | ***F*** | ***p*** |
| --- | --- | --- | --- | --- | --- |
| Sex | 0.002 | 1 | 0.002 | 0.02 | 0.88 |
| Captivity status | 4.87 | 1 | 4.87 | 62.18 | <0.0005 |
| Age | 0.17 | 3 | 0.06 | 0.72 | 0.54 |
| Sex : captivity status | 0.13 | 1 | 0.13 | 1.66 | 0.20 |
| Sex : age | 0.11 | 3 | 0.04 | 0.45 | 0.72 |
| Captivity status : age | 0.42 | 1 | 0.42 | 5.31 | 0.02 |
| Sex : captivity status : age | 0.02 | 1 | 0.02 | 0.26 | 0.61 |
| Error | 5.17 | 66 | 0.08 |  |  |

**Table D4. Total serum fatty acids.**

| **Source** | **Sum of squares** | ***df*** | **Mean square** | ***F*** | ***p*** |
| --- | --- | --- | --- | --- | --- |
| Sex | 195669.83 | 1 | 195669.83 | 0.16 | 0.69 |
| Captivity status | 15730023.88 | 1 | 15730023.88 | 13.06 | 0.001 |
| Age | 4867415.22 | 3 | 1622471.74 | 1.35 | 0.27 |
| Sex : captivity status | 1927443.44 | 1 | 1927443.44 | 1.60 | 0.21 |
| Sex : age | 2105389.80 | 3 | 701796.60 | 0.58 | 0.63 |
| Captivity status : age | 2175126.54 | 1 | 2175126.54 | 1.81 | 0.18 |
| Sex : captivity status : age | 413071.84 | 1 | 413071.84 | 0.34 | 0.56 |
| Error | 79478845.05 | 66 | 1204224.93 |  |  |

|  |
| --- |
